# Artificial Intelligence-Assisted Muscular Ultrasonography for Assessing Inflammation and Muscle Mass in Patients at Risk of Malnutrition

**DOI:** 10.3390/nu17101620

**Published:** 2025-05-09

**Authors:** Juan José López-Gómez, Lucía Estévez-Asensio, Ángela Cebriá, Olatz Izaola-Jauregui, Paloma Pérez López, Jaime González-Gutiérrez, David Primo-Martín, Rebeca Jiménez-Sahagún, Emilia Gómez-Hoyos, Daniel Rico-Bargues, Eduardo Jorge Godoy, Daniel A. De Luis-Román

**Affiliations:** 1 Servicio de Endocrinología y Nutrición, Hospital Clínico Universitario de Valladolid, 47003 Valladolid, Spain; 2 Centro de Investigación en Endocrinología y Nutrición, Universidad de Valladolid, 47003 Valladolid, Spain; 3 DAWAKO Medtech S.L., Parc Cientific de la Universitat de Valencia, 46980 Paterna, Spain; 4 Técnicas Avanzadas de Desarrollo de Software Centrado en la Persona, Departamento de Informática, Universitat de Valencia, 46010 Valencia, Spain

**Keywords:** inflammation, C-reactive protein, muscular ultrasonography, artificial intelligence, disease-related malnutrition

## Abstract

Background: Malnutrition, influenced by inflammation, is associated with muscle depletion and body composition changes. This study aimed to evaluate muscle mass and quality using Artificial Intelligence (AI)-enhanced ultrasonography in patients with inflammation. Methods: This observational, cross-sectional study included 502 malnourished patients, assessed through anthropometry, electrical bioimpedanciometry, and ultrasonography of the quadriceps rectus femoris (QRF). AI-assisted ultrasonography was used to segment regions of interest (ROI) from transversal QRF images to measure muscle thickness (RFMT) and area (RFMA), while a Multi-Otsu algorithm was used to extract biomarkers for muscle mass (MiT) and fat mass (FatiT). Inflammation was defined as C-reactive protein (CRP) levels above 3 mg/L. Results: The results showed a mean patient age of 63.72 (15.95) years, with malnutrition present in 82.3% and inflammation in 44.8%. Oncological diseases were prevalent (46.8%). The 44.8% of patients with inflammation (CRP > 3) exhibited reduced RFMA (2.91 (1.11) vs. 3.20 (1.19) cm^2^, *p* < 0.01) and RFMT (0.94 (0.28) vs. 1.01 (0.30) cm, *p* < 0.01). Muscle quality was reduced, with lower MiT (45.32 (9.98%) vs. 49.10 (1.22%), *p* < 0.01) and higher FatiT (40.03 (6.72%) vs. 37.58 (5.63%), *p* < 0.01). Adjusted for age and sex, inflammation increased the risks of low muscle area (OR = 1.59, CI: 1.10–2.31), low MiT (OR = 1.49, CI: 1.04–2.15), and high FatiT (OR = 1.44, CI: 1.00–2.06). Conclusions: AI-assisted ultrasonography revealed that malnourished patients with inflammation had reduced muscle area, thickness, and quality (higher fat content and lower muscle percentage). Elevated inflammation levels were associated with increased risks of poor muscle metrics. Future research should focus on exploring the impact of inflammation on muscles across various patient groups and developing AI-driven biomarkers to enhance the diagnosis, monitoring, and treatment of malnutrition and sarcopenia.

## 1. Introduction

Malnutrition is a disease that may be caused by an unusually reduced intake or assimilation of nutrients, but the most recent definition considers the inflammatory state to be both a cause and a consequence of this entity [1]. The Global Leadership Initiative on Malnutrition (GLIM) has adopted an approach based on inflammation for the diagnosis of disease-related malnutrition as an etiologic criterion. This criterion can be based on the presence of acute or chronic disease, infection, or injury, but the management of C-reactive protein (CRP) as a key biomarker can be used when inflammatory components are uncertain [2]. A diagnosis using GLIM criteria incorporating chronic inflammation has a stronger predictive value than the original GLIM criteria for both short- and long-term prognosis in cancer patients [3]. The predictive capability of a GLIM diagnosis for survival appears to be influenced by the specific etiologic criteria applied [4]. In these patients, muscle depletion and body composition are strong predictors of cancer survival in patients with different body mass index values [5].

Inflammation is a biologic response of the body to infection, injury, and other harmful stimuli. Although crucial for tissue defense and repair, chronic, low-grade inflammation can have significant adverse effects on body composition, especially on muscle health [6]. The essential factors contributing to skeletal muscle atrophy in inflammation are proteolysis, diminished protein synthesis, and impaired regeneration of muscle fibers. This condition can result as a direct effect of activating receptor-mediated signaling pathways within the muscles, such as nuclear factor-kB, Janus-activated kinase/signal transducer and activator of transcription (JAK/STAT), and p38 mitogen-activated protein kinase (p38MAPK); these factors are related to proteolysis, decreases in protein synthesis, and cellular senescence [7]. It can also result as an indirect effect of dysregulation of the hypothalamic–pituitary–adrenal axis because inflammatory cytokines can disrupt negative feedback regulation in the hypothalamus and pituitary, causing hyperactivation of the axis. This results in excessive steroid levels in the bloodstream, triggering muscle atrophy. Another indirect effect of muscle atrophy consists of altered fat metabolism control through inflammatory factors such as IL-6, TNF-a, and IL-1B. These factors prevent inadequate lipoprotein metabolism after increased lipolysis due to fasting and nutritional deterioration [7].

Chronic inflammation may play a role in the decline of muscle strength, which is the main criterion for diagnosing sarcopenia. It seems that high levels of C-reactive protein (CRP) are associated with lower muscle strength, as highlighted in a systematic review by Shokri-Mashhadi et al. [8]. Several studies on patients with chronic diseases have shown that a proinflammatory state with higher levels of CRP is associated with lower handgrip strength, such as that from Kim et al. on a general population from the Korea National Health and Nutrition Survey [9]. Muscle quality plays a crucial role in muscle function. In this context, inflammation can induce metabolic dysfunction, leading to fat infiltration within the muscle and resulting in myosteatosis. This condition can be produced by a differentiation of stem cells into fibro-adipogenic precursors and then into intramuscular adipose tissue (IMAT); another mechanism, in nutrient overload, could be associated with the release of adipose-derived stem cells from subcutaneous adipocytes, which infiltrate the muscle [10]. IMAT accumulation is associated with a decrease in muscle mass and function (muscle strength and physical performance) [11].

Inflammation can have harmful effects on muscle tissue, which can manifest in various ways. The imaging diagnosis of muscles can be performed using several techniques. In patients with cancer cachexia, high weight loss, a low muscle index, and reduced muscle attenuation, as determined by computed tomography, were associated with decreased survival, independent of body mass index (BMI) classification [5]. CT images can show us an adequate measure of muscle depletion in these patients but also an accurate measure of muscle quality and the presence of myosteatosis, which is highly related to muscle function and prognosis [10]. Nevertheless, this technique is expensive, requires exposure to X-rays, and is time-consuming. For these reasons, CT cannot be used as a rutinary method in all patients to diagnose malnutrition or sarcopenia. Bioelectrical impedance analysis is an easy, cost-effective technique to evaluate the inflammatory status of patients, and the phase angle is related to prognosis in patients with disease-related malnutrition [12]. This technique allows us to estimate muscle mass, but it cannot directly evaluate the muscle mass and quality in a direct manner, with potential overestimations in muscle mass [13].

Muscular ultrasonography is a straightforward and highly cost-effective technique. It does not involve X-ray radiation, is quick to perform, and eliminates the need for additional patient visits, as it can be performed during a clinical consultation [14]. Muscular ultrasonography is a good technique to evaluate muscle mass in sarcopenia and malnutrition, which are related to the cellularity assessed by bioelectrical impedanciometry [15], and handgrip strength and sarcopenia diagnosis [16]. This technique is also a useful method to evaluate muscle quality, with a relationship between the anteroposterior and transversal axis (X-Y index) of a transversal image of the quadriceps rectus femoris (QRF) and echogenicity; we observe that a lower X-Y index and a lower echogenicity are related to poor muscle quality, with correlations with phase angle and handgrip strength [15]. Muscle echogenicity showed moderate to strong correlations with skeletal muscle density measured by CT, intramuscular adipose tissue (IMAT) assessed by MRI, and IMAT, as well as extramyocellular lipids evaluated by Magnetic Resonance Spectroscopy [10]. Muscle ultrasound can assess changes in muscle echogenicity and volume, facilitating the early detection of inflammation damage to muscles. For these reasons, this technique can serve as a useful tool to diagnose malnutrition and sarcopenia and to monitor treatments for these diseases [17].

The integration of AI into imaging techniques dedicated to body composition—such as muscle and fat ultrasound at various sites, CT, and MRI—has enabled significant advances in automated image segmentation. By leveraging cutting-edge methods like machine learning, deep learning, and neural networks, these systems can efficiently analyze various body components (including fat, muscle, and other structures) and derive both quantitative and qualitative parameters. Moreover, these tools utilize pattern recognition and large-scale data analysis to optimize the processing of medical images. In addition to speeding up the extraction of quantitative features, they also assess qualitative aspects, effectively transforming images into valuable data that enhance clinical decision-making. This approach aligns with the innovative concept of radiomics, combining AI’s analytical power to enable more precise diagnoses and personalized treatments in body composition analysis [18].

The use of Artificial Intelligence (AI)-based tools for ultrasound imaging analysis can help us to decrease measure variability [19] and for a better evaluation of muscle quality [20]. These tools can help us to obtain a huge amount of information from ultrasound images with little waste of time. This study aimed to assess the impact of the proinflammatory state on muscle quantity and quality, as determined by AI-assisted ultrasonography, in patients at risk of malnutrition.

## 2. Materials and Methods

### 2.1. Study Design and Eligibility Criteria

This is an observational transversal study that included patients aged more than 18 years old at risk of malnutrition. These patients were recruited from the Endocrinology and Nutrition Service of the Clinic University Hospital of Valladolid, Spain, over the period from January 2021 to September 2024. The inclusion criteria were patients at risk of malnutrition and more than 18 years old; the exclusion criteria were chronic kidney disease stage IV or higher, uncontrolled liver disease, oncologic patients in terminal phases, and those who refused to signed the informed consent.

**Figure 1:**
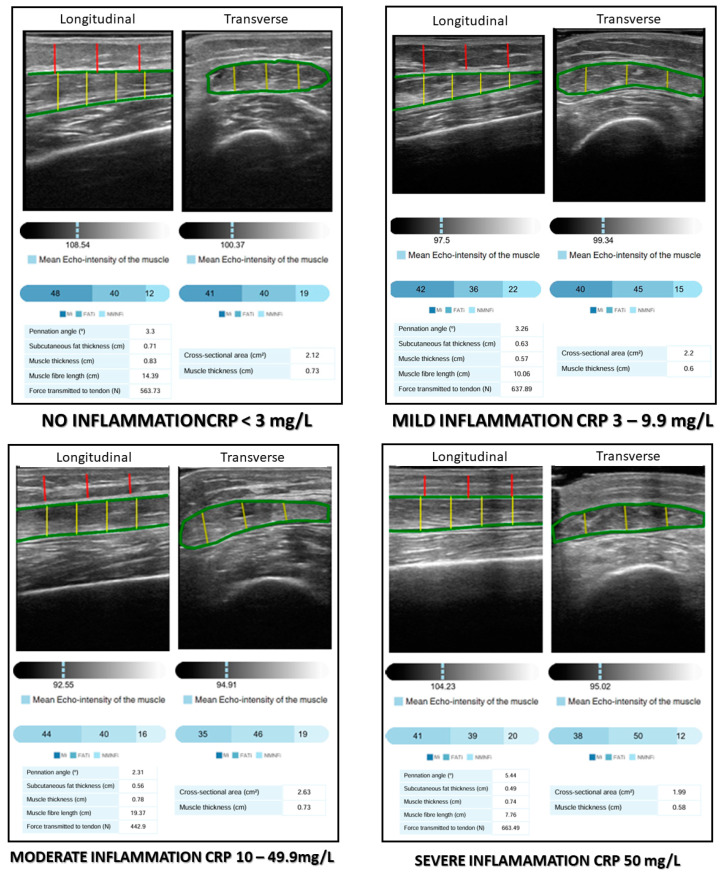
AI-assisted rectus femoris ultrasonography and degrees of inflammation. MiT: muscle index; FATiT: fat index; NMNFiT: no muscle no fat index; CRP: C-reactive protein.

The patients were assessed based on their nutritional history, anthropometric measures, electrical bioimpedanciometry, quadriceps rectus femoris muscular ultrasonography, and biochemistry analytics with inflammation and nutritional biomarkers. The images obtained through ultrasonography were processed using an Artificial Intelligence (AI)-based ultrasound imaging system (PIIXMEDTM; DAWAKO MedTech; Valencia, Spain) (Figure 1).

This study was conducted in accordance with the Declaration of Helsinki and approved by the Ethics Committee for Clinical Research of the Health Council of Valladolid Areas (protocol code PI 22-2907 on 13 October 2022). We obtained signed informed consent from all eligible participants before their recruitment.

### 2.2. Variables

#### 2.2.1. Anthropometric Measures

Anthropometric data, including height (m), body weight (kg), and body mass index (BMI, kg/m^2^), were collected. Body weight was measured using a scale with 100 g accuracy (0.1 kg; Seca, Birmingham, United Kingdom), with patients in an unclothed state. Height was obtained using a stadiometer (Seca, Birmingham, United Kingdom), with the patients standing upright. BMI was determined using the formula body weight/(height × height) (kg/m^2^).

The percentage of body weight loss was calculated using the following equation:

[(Usual body weight (kg) − Current body weight (kg))/Usual body weight] × 100

Additionally, upper arm circumference (AC, cm) and calf circumference (CC, cm) were measured. These measurements were taken using a precision measuring tape with a 0.01 m accuracy. AC was measured on the right arm at the midpoint between the acromion process and the olecranon process, with the arm hanging relaxed at the side. The CC was measured at the point of maximum girth of the right calf, with the participant standing upright and weight evenly distributed.

#### 2.2.2. Electrical Bioimpedanciometry (BIA)

This technique was performed with a bioimpedanciometer (BIA 101 Anniversary; EFG Akern, Pisa, Italy) between 8:00 and 10:00, after an overnight fast and 15 min in the supine position. The device featured a signal generator that produced an alternating current of 0.8 mA at 50 kHz, with electrodes positioned on the back of the right hand and foot. The variables obtained were raw electrical data: reactance (ohm); resistance (ohm), and phase angle (°). Total body water (TBW) was estimated using the Lukaski formula for the analysis of hydration status [21]. The appendicular skeletal muscle index (ASMI) to diagnose low muscle mass by BIA was estimated with Sergi’s Formula [21] in order to diagnose malnutrition. Malnutrition and sarcopenia were identified based on ASMI thresholds of <7 kg/m^2^ in men and <5.5 kg/m^2^ in women [1].

#### 2.2.3. AI-Based Muscular Ultrasonography

Muscle ultrasonography of the quadriceps rectus femoris (QRF) was carried out on the dominant lower extremity with a 10–12 MHz probe and a multifrequency linear matrix (Mindray Z60, Madrid, Spain). The measurements of ultrasonography were made with the patient in a supine position. The probe was placed perpendicular to the transverse axis of the dominant leg (lower third of the distance between the iliac crest and the upper border of the patella) [22].

The images obtained through ultrasonography were processed using an AI-based ultrasound imaging system (PIIXMEDTM; DAWAKO MedTech; Valencia, Spain). This cloud-based platform employs a convolutional neural network (CNN) featuring a U-net architecture, which is designed for rapid and precise image segmentation. The U-net architecture has demonstrated superior performance compared to traditional sliding-window convolutional networks. This program uses a modified U-net architecture specifically tailored for automated musculoskeletal segmentation. The PIIXMEDTM system allows for feature extraction in 2D for conventional B-Mode US imaging and can calculate single values per feature for a region of interest (ROI). From the identified features and application of various algorithms, diverse biomarkers were extracted and processed to analyze the ROI’s morphological architecture, muscle quality based on echogenicity, and different matrix-based biomarkers of texture [19]. The muscle mass parameters evaluated were rectus femoris muscle area (RFMA) in cm^2^ and rectus femoris muscle thickness (RFMT) in cm, indicating the cross-sectional muscle area and thickness within the ROI of the muscle belly. Subcutaneous fat thickness (SFT) measured the thickness of subcutaneous adipose tissue in the longitudinal section. It is important to note that the tool was trained on a comprehensive dataset comprising thousands of images from various muscle groups, including the rectus femoris. These images were collected from healthy individuals, athletes, and patients with a range of pathological conditions, ensuring robust and diverse training data for accurate segmentation.

The validation of the AI-based ultrasound imaging system was performed in a cohort of 100 patients with disease-related malnutrition (DRM). A skilled sonographer obtained ultrasound images of the rectus femoris, which were evaluated using both conventional methods and the automated PIIXMED^TM^ system. The results demonstrated high consistency and minimal bias between the two methods, confirming that the AI-based tool provides accurate and reliable quantitative assessments of muscle parameters in DRM patients [19].

Muscle quality was assessed based on the pennation angle (in degrees), defined as the angle between the muscle fibers and the lower aponeurosis. A higher pennation angle indicates a greater capacity for force production [23]. Muscle quality indexes were determined using a multi-thresholding algorithm (i.e., Multi-Otsu algorithm), which is based on grey intensity levels within the image and defines thresholds to separate the pixels of an ultrasound image into different classes. The Multi-Otsu algorithm is an extension of the Otsu method, which is used in image processing to segment an image into multiple intensity levels. While the original Otsu method divides an image into two classes (for example, background and object), the Multi-Otsu algorithm allows the image to be segmented into more than two classes, which is useful for images with multiple regions of interest. The algorithm works by finding the optimal thresholds that minimize the within-class variance and maximize the between-class variance for each segmentation level. This is achieved through a statistical analysis of the image histograms. This algorithm determines the threshold values for three categories measured in a transversal image: muscle index (MiT) as a percentage of muscular tissue in the transversal ROI; fat index (FATiT) as a percentage of fat in the transversal ROI; and the no muscle no fat index (NMNFiT) as the percentage of other complex structures such as collagen, connective tissue, and fibrosis in the transversal ROI [24]. These indexes were represented as percentages from the ROI (Figure 1).

#### 2.2.4. Functional Status

Muscle function was evaluated by measuring handgrip strength using a JAMAR^®^ dynamometer (Basel, Switzerland). The patients were seated with their dominant arm positioned at a 90° angle relative to the forearm during the test. For sarcopenia diagnosis, we applied the European Working Group on Sarcopenia in Older People (EWGSOP2) criteria [25]. Specifically, a sarcopenia diagnosis required both reduced handgrip strength (defined as <27 kg for men and <16 kg for women) and low muscle mass (defined as an ASMI of <7 kg/m^2^ for men and < 5.5 kg/m^2^, as determined by BIA). Patients with decreased handgrip strength but normal muscle were classified as having probable sarcopenia or dynapenia.

#### 2.2.5. Nutritional Blood Biomarkers

The biochemical blood parameters were made in the usual control analysis (Cobas c-711 autoanalyzer (Roche Diagnostics, Basel, Switzerland)). The blood biomarkers measured were C-reactive protein (CRP) (mg/L); albumin (g/dL); and prealbumin (g/dL). We have evaluated new indexes that combine inflammatory markers as CRP and serum proteins; these indexes were CRP/albumin and CRP/prealbumin (14).

#### 2.2.6. Nutritional Diagnosis

- Malnutrition diagnosis: A malnutrition diagnosis was made through the Global Leadership Initiative on Malnutrition (GLIM) criteria. Patients must have one phenotypic criterion and one etiologic criterion [1].
  - Phenotypic criteria: BMI and percentage of weight loss was assessed by anthropometric measures. Loss of muscle mass was assessed by BIA-estimated ASMI with Sergi’s Formula (ASMI < 7 kg/m^2^ in men and ASMI < 5.5 kg/m^2^ in women).
  - Etiologic criteria: Reduced food intake was assessed by a semiquantitative method in patient’s anamnesis, and disease burden/inflammation was considered based on their C-reactive protein levels. Low food intake was considered as below 75% of usual intake, and inflammation was considered with a level of C-reactive protein over 3 mg/L.
- Inflammation diagnosis: The degree of inflammation was assessed as the recommendation made in the Cederholm et al. Delphi study for guidance of etiologic criterion in GLIM [2]. We consider the following degrees of inflammation: no inflammation (CRP < 3 mg/L); mild inflammation (CRP = 3–9.9 mg/L); moderate inflammation (CRP = 10–49.9 mg/L); and severe inflammation (CRP > 50 mg/L).
- AI-based muscle ultrasonography diagnosis: In order to analyze the effect of inflammation on muscle parameters, we considered a quantitative method to diagnose low muscle mass and low muscle quality with the median values of our sample:
  - Muscle mass: A diagnosis of low muscle mass by US ultrasonography was made based on RFMA and RFMT. Low muscle mass was considered positive by RFMA with a value lower than the median of the sample (3.5 cm^2^ in men and 2.7 cm^2^ in women). Low muscle mass was considered positive by RFMT with a value lower than the median of the sample (1.06 cm in male and 0.9 cm in women).
  - Muscle quality: A diagnosis of low muscle quality by US ultrasonography was made based on low MiT and high FATiT. Muscle quality was considered low by MiT with a value lower than the median of the sample (< 47% in men and <44% in women). Muscle quality was considered low by FATiT with a value higher than the median of the sample (>36% in men and women).

For scoring purposes, one point was assigned when low muscle mass was detected (as defined by the median values for RFMA and RFMT), while high muscle mass received a score of zero. Similarly, for the region of interest (ROI), a score of one was given when a low percentage of muscle tissue was observed, and a score of zero was assigned in the presence of a high percentage of fat infiltration.

### 2.3. Data Analysis

A statistical analysis was conducted using the SPSS 15.0 software package (SPSS Inc. v.23, Chicago IL, USA), officially licensed by the University of Valladolid. A normality test for continuous variables was performed using the Kolmogorov–Smirnov test. Normally distributed continuous variables are expressed as means (standard deviation), and non-normally distributed continuous variables are expressed as medians (interquartile range). Qualitative variables are represented by the number and percentage of the total sample.

Differences between parametric continuous variables were analyzed using the unpaired Student’s *t*-test, while differences between non-parametric variables were analyzed using the Mann–Whitney U test. If comparisons among more than two groups were necessary, the ANOVA test (with the Bonferroni post hoc test) was employed. A correlation analysis was performed to evaluate the relationship between quantitative variables. A binary logistic regression was conducted in a multivariate analysis to assess the relationship of the variables with the prognosis. *p* values under 0.05 were considered statistically significant.

## 3. Results

### 3.1. Sample Description

A total of 502 patients at risk of malnutrition were recruited for this study; 289 (57.6%) of the patients were women. The mean age of the patients was 63.72 (15.95) years; 274 (54.6%) of patients were older than 65 years old.

**Figure 2:**
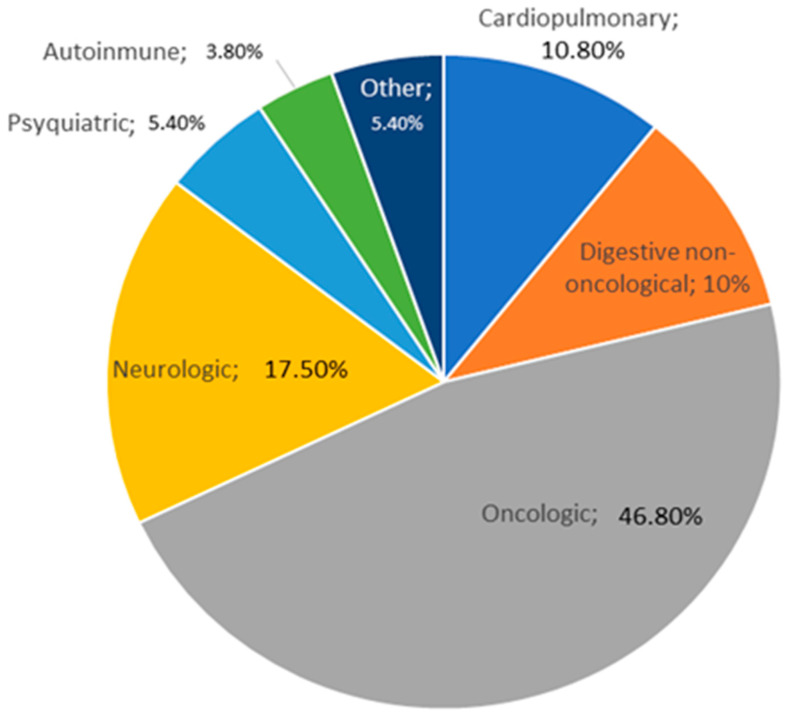
Distribution of pathologies in the population sample.

Most of the patients had oncological diseases, 235 (46.8%). Other conditions included neurological disorders (17.5%), cardiopulmonary issues (10.8%), digestive non-oncological diseases (10%), psychiatric disorders (5.4%), autoimmune diseases (3.8%), and other pathologies (5.4%). The distribution of different diseases which may lead to malnutrition are represented in Figure 2.

**Figure 3:**
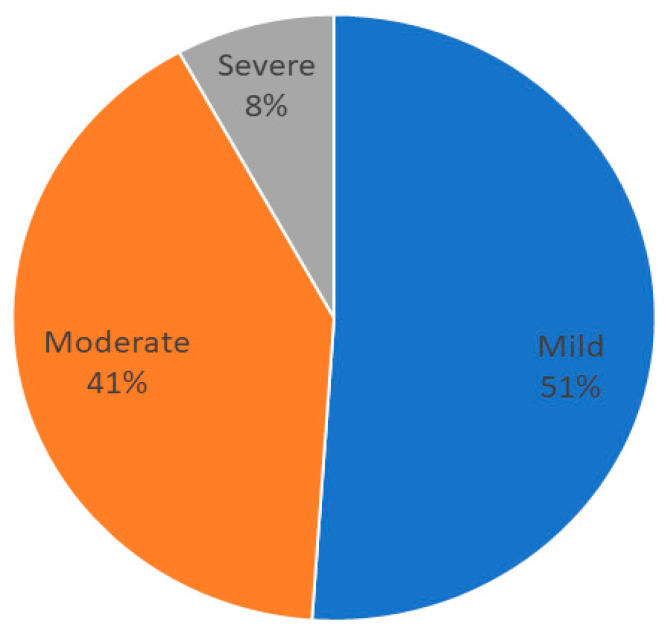
Distribution of grades of inflammation in patients.

The median value of C-reactive protein (CRP) was 2.19 (1–7.85) mg/L; 225 patients (44.8%) presented with inflammation. The percentage of patients with mild (3–9.9 mg/L), moderate (10–50 mg/L), or severe inflammation (>50 mg/L) based on CRP are shown in Figure 3.

The median value of CRP to prealbumin ratio (CRP/prealbumin) was 0.10 (0.05–0.38), and the median value of CRP to albumin ratio (CRP/albumin) was 0.51 (0.24–1.95).

Malnutrition was present in 413 (82.3%) patients 258 (51.4%) patients had severe malnutrition. If we only consider inflammation as an etiological criterion, 322 (64.1%) patients presented with malnutrition. Sarcopenia was present in 131 (26.1%) patients.

The differences in the variables of anthropometry, impedanciometry, and muscular ultrasonography between sex are shown in Table 1.

### 3.2. AI-Assisted Muscular Ultrasonography in Malnutrition and Inflammation

The patients with inflammation (CRP > 3) had less rectus femoris muscle area (RFMA) (CRP > 3: 2.91 (1.11) cm^2^; CRP < 3: 3.20 (1.19) cm^2^; *p* < 0.01) and muscle thickness (RFMT) (CRP > 3: 0.94 (0.28) cm; CRP < 3: 1.01 (0.30) cm; *p* < 0.01) measured by AI-assisted muscular ultrasonography. In terms of muscle quality, the transversal ROI of the rectus femoris in patients with inflammation had less quality (less percentage of muscle (MiT) (CRP > 3: 45.32 (9.98%); CRP < 3: 49.10 (1.22%); *p* < 0.01) and more percentage of fat (FATiT) (CRP > 3: 40.03 (6.72%); CRP < 3: 37.58 (5.63%); *p* < 0.01)).

The differences in AI-assisted ultrasonography and other body composition variables depending on the degree of inflammation are shown in Table 2. Patients with a high grade of inflammation had a decrease in phase angle, RFMT, RFMA and MiT and an increase in FATiT. However, the patients with a high grade of inflammation had a higher age.

### 3.3. Relationship Between Muscle Mass and Quality with Inflammation

#### 3.3.1. Muscle Mass

CRP had an inverse correlation with rectus femoris area and no relationship with the other parameters of ultrasonography muscle mass (SFT or RFMT) (Table 3).

**Figure 4:**
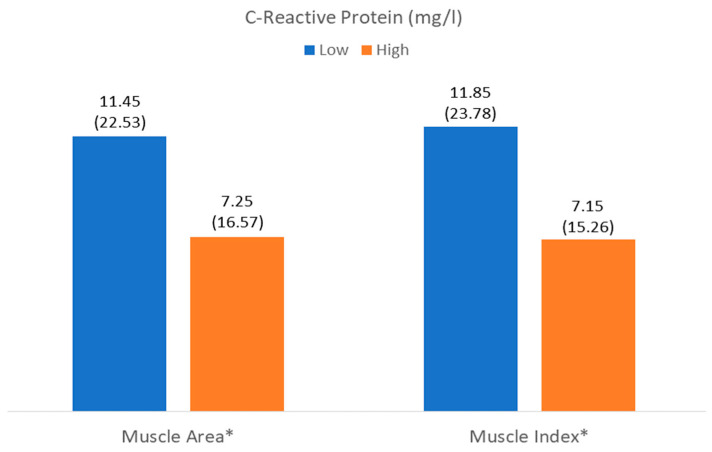
Differences in C-reactive protein related to muscle mass (muscle area) and muscle quality (muscle index (Mi)). * *p* < 0.05.

**Figure 5:**
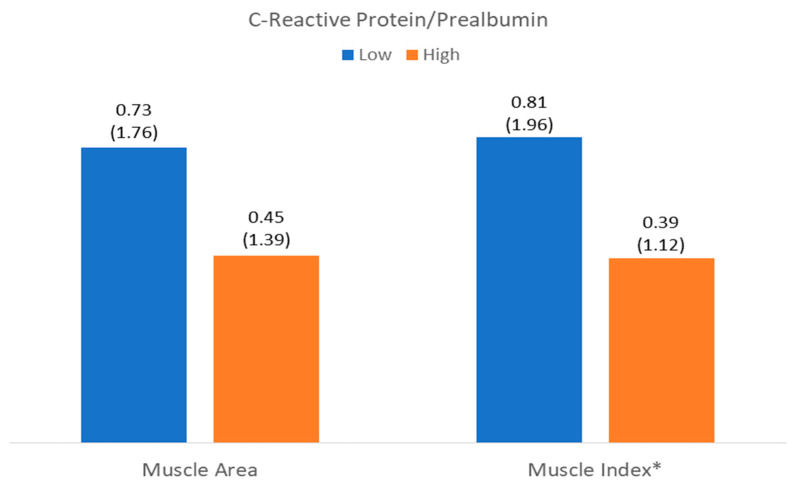
Differences in C-reactive protein to prealbumin ratio related to muscle mass (muscle area) and muscle quality (muscle index (Mi)). * *p* < 0.05.

The number of patients with low muscle mass by area (LowMMUSArea) was 261 (52%), and that with low muscle mass by muscle thickness (LowMMUSthickness) was 250 (49.8%). There was a higher level of C-reactive protein in patients with LowMMUSArea, but there were no differences in the other parameters or in the other variables in patients with LowMMUSthickness (Figure 4 and Figure 5).

There was a risk of LowMMUSArea in patients with a high grade of inflammation adjusted by age and sex; there was no risk of LowMMUSthickness in patients with a high grade of inflammation adjusted by age and sex (Table 4).

#### 3.3.2. Muscle Quality

CRP had an inverse correlation with MiT and a direct correlation with FATiT and NMNFiT but no relationship with pennation angle (Table 3). There was also a relationship of these AI-generated variables (MiT, FATiT, and NMNFiT) with CRP to prealbumin ratio and CRP to albumin ratio (Table 3).

The number of patients with low muscle percentage in the ROI (LowMiT) was 244 (48.60%) and that with high fat percentage in the ROI (HighFATiT) was 263 (52.40%).

There was a higher level of CRP and CRP/prealbumin in patients with LowMiT, but there were no differences in the other variables such as HighFATiT (Figure 4 and Figure 5).

There was a risk of LowMiT in patients with a high grade of inflammation and sarcopenia adjusted by age and sex; there was also a high risk of HighFATiT in patients with a high grade of inflammation adjusted by age and sex (Table 5).

## 4. Discussion

This study aims to assess the relationship between inflammation and the deterioration of muscle mass and quality using an AI-based analysis of quadriceps rectus femoris ultrasonography. This system evaluates muscle mass and echogenicity by differentiating the percentage of muscle and fat within the measured region of interest (ROI). The main results observed were low muscle mass (muscle area and muscle thickness) and a low muscle quality (low muscle percentage and high fat percentage) in patients with a high grade of inflammation. There was a high risk of low muscle mass and low muscle quality in patients with CRP over 3 mg/L adjusted by sex and age.

### 4.1. Sample Characteristics

Disease-related malnutrition and inflammation are related as cause and consequence. In fact, inflammation is considered one of the etiologic criteria for disease burden in the GLIM criteria, and its measurement is a potential challenge in diagnosing malnutrition and evaluating the risk of complications in these patients [26]. In our sample, a significant proportion of the studied patients (82.3%) were malnourished, and 44.8% had inflammation. Most of the patients of our sample had an oncologic pathology that is a condition that can lead to an inflammatory state, and a diagnosis based on the GLIM criteria based on this alteration had more predictive value to predict survival, as Xie et al. showed in their study [3]. In our sample, not all of the patients met the inflammation criteria: 64.1% had a malnutrition diagnosis with the inflammation criteria. The grade of inflammation was mild to moderate in most patients, with a small percentage of patients with severe inflammation. This classification of inflammation degree by C-reactive protein values was carried out following the recommendations by ESPEN [27]. The use of these markers and this stratification may help us to assess the risk of comorbidities and mortality of the patient [3].

The descriptive evaluation of nutritional assessment showed differences between sexes in all parameters of body composition (anthropometry, bioimpedanciometry, and ultrasonography) including measures of muscle mass and muscle quality, without differences in terms of inflammation between sexes. These sex-related differences are consistent with those reported in previous studies. Differences in muscle mass evaluated by ultrasonography were also reported in the DRECO study by de Luis et al. in patients with disease-related malnutrition [16]. Conversely, differences in muscle quality assessed by ultrasonography (echogenicity) are less well established in the literature; however, one previous study carried out by our group using this IA tool found that women had lower values of MiT and higher values of FaTiT in the ROI [20].

### 4.2. Muscle Mass and Quality and Inflammatory Status

Inflammation has a great impact on body composition, with an influence on fat mass, on total body water, and specially in muscle mass. Variations in compartmental changes complicate anthropometric assessment due to interference from body fluids, making clear differences observable only in severe cases of malnutrition [28]. In our sample, patients with higher levels of inflammation had higher values of BMI and patients with mild inflammation had higher levels of arm circumference than those without inflammation; these conditions are related to low sensitivity of these methods in evaluating malnutrition. Bioimpedanciometry revealed a reduction in reactance and phase angle in patients with inflammation, with the degree of this reduction aligning with the severity of the inflammation. These results are linked to alterations in cell membranes caused by inflammation-induced damage or congestion, which may contribute to disease-related malnutrition. A low phase angle has been demonstrated as a biomarker of inflammation and a predictor of prognosis in patients with disease-related malnutrition [12]. In our sample, patients with higher degrees of inflammation were older. Aging may be associated with an increase in the levels of reactive oxygen species, DNA damage, and cell apoptosis, which could be related to an increase in inflammation [29]. On the other hand, aging is associated with a decrease in muscle mass and function, which can lead to the development of sarcopenia regardless of the presence of disease-related malnutrition [30]. However, the combination of aging and the presence of chronic comorbidities is associated with an increased risk of sarcopenia, both in patients with normal body weight and those with obesity [31].

Muscular ultrasonography is an effective tool for assessing muscle mass and quality in patients with disease-related malnutrition. In such patients or those with sarcopenia, ultrasonography often reveals reduced muscle volume, reflecting muscle health and metabolic activity. Alterations in muscle architecture may signify impaired strength and endurance, while increased echogenicity is associated with intermuscular fat infiltration and fibrosis [17]. These conditions can be related to inflammatory processes and can show us an alteration in muscles previous to the changes in muscle function and can help us to implement treatments to improve these pathologies [10]. In our sample, patients with inflammation exhibited lower muscle mass and signs of poor muscle quality, including a lower muscle mass percentage; a higher fat mass percentage; and the presence of non-muscle, non-fat structures such as fibrosis. These conditions were more pronounced in patients with a higher degree of inflammation. In our sample, handgrip strength did not differ significantly between inflammation groups. However, we observed a moderate correlation between handgrip strength and ultrasound-assessed muscle mass, and a weak correlation with muscle quality variables. This suggest that a decline in muscle quality may occur early in the presence of inflammation, preceding detectable functional impairment.

The correlations observed in this study highlight a complex interplay between inflammatory markers, hydration status, and muscle composition assessed by ultrasonography. Inflammatory markers tend to be associated with a slight reduction in muscle mass and quality. This condition can be related to the heterogeneity of the sample, which includes many types of diseases with different physiopathologies. In contrast, a higher phase angle (marker of cellular integrity) is linked to better muscle parameters and a lower fat infiltration with a moderate correlation. Similarly, total body water demonstrates relationships that support the idea that improved hydration correlates with better muscle characteristics. While many of these correlations are modest, their statistical significance points to meaningful associations that could contribute to a better understanding of the muscle–inflammation–hydration nexus in clinical populations [12]. These findings underscore the need to consider both inflammatory and hydration statuses when assessing muscle quality, and they may provide valuable insights for developing interventions aimed at improving muscle characteristics in patients with altered inflammatory states.

Muscle mass was assessed based on the rectus femoris muscle area and rectus femoris muscle thickness. We observed higher values of muscle mass parameters in patients with lower values of CRP; this relation was not observed in the CRP/albumin ratio. This relationship between inflammation and quadriceps rectus femoris mass was evaluated by Battaglia et al., and they observed a relationship between QRF thickness and Malnutrition Inflammation Score (MIS) in patients with chronic dialysis [32]; in our study, we found no relation with muscle thickness, but there was a relationship with muscle mass assessed based on muscle area. In patients with a high grade of inflammation such as those with sepsis, RFMA showed moderate correlation with strength, better than muscle thickness, in the study of Palakshappa et al. [33]. These differences can be related to the type of pathology and fluid status, as Stanley et al. observed, with a direct correlation between muscle quantity and fluid status measured by bioimpedanciometry [34]. On the other hand, the association of muscle parameters with biochemical biomarkers of inflammation has a relationship with ICU-acquired weakness, as Lei et al. had observed in their study [35].

The primary differences in muscle quality associated with inflammation identified so far include an increase in muscle echogenicity. This change reflects the extent of muscle dehydration and degeneration, correlating with the presence of fibrous tissue and the accumulation of fat within the muscle [10,17]. In our study, patients with inflammation had a higher percentage of fat and other non-muscular tissues in the ROI and a decrease in muscular tissue. Yoshida et al. observed that muscle echogenicity was a surrogate marker of inflammation in idiopathic inflammatory myopathies at early stages [36]. Muscle echogenicity has shown be a useful tool in predicting myofiber necrosis and fascial inflammation in critical patients [37,38]; for this reason, the use of echogenicity in the assessment of muscle quality can help us to act before muscle decline.

### 4.3. Clinical Implications

AI-enhanced muscle ultrasonography represents a promising advancement in evaluating muscle mass and quality. Automatic segmentation can reduce interobserver variability and time to capture the ROI [18]. On the other hand, the use of specific deep learning methods can be adapted to the special characteristics of every image. These techniques generate novel biomarkers that can be extrapolated, and they can be used to diagnose and monitoring treatment. Biomarkers have been used to assess muscle quality, known as the muscle index (MiT), and muscle mass and its changes in patients in medical nutrition treatment with oral nutrition supplements, known as the fat index (FaTiT) [19]. The parameters of muscle quality obtained by this AI tool has shown a relationship with the state of inflammation in the present study, being consistent with the existing evidence showing an increase in fat percentage and a decrease in muscle percentage [17].

Automation in imaging-based body composition segmentation not only speeds up the analysis process but also standardizes the extraction of quantitative tissue data, thereby facilitating the use of these biomarkers for diagnosing and monitoring nutritional medical treatments [18]. In parallel, muscular ultrasonography has proven to be an effective tool for assessing muscle mass and quality in patients with disease-related malnutrition or sarcopenia. This technique often reveals reduced muscle volume, alterations in muscle architecture that may signal impaired strength and endurance, and increased echogenicity associated with intermuscular fat infiltration and fibrosis [17]. Such conditions, which are linked to inflammatory processes, can serve as early indicators of muscle alterations before overt functional decline occurs, thus allowing timely interventions [10]. In our sample, patients with inflammation exhibited lower muscle mass and poorer muscle quality—as evidenced by a reduced muscle mass percentage and a higher fat mass percentage—with these alterations being more pronounced in those with higher inflammation levels. These muscle characteristics have been related also with some clinical issues in patients with oncological disease, with a recent study having shown that patients with higher levels of muscle area and thickness measured by AI technology have been related to a better quality of life measured by EuroQol-5D [39].

Despite the promising potential of these automated and AI-assisted imaging techniques, standardization of measurement protocols remains necessary for their proper integration into everyday clinical practice. Advances in AI-driven medical image segmentation are expected to further enhance the speed, accuracy, and reproducibility of these assessments, thereby offering significant clinical implications for early diagnosis, optimized treatment strategies, and improvement patient care.

### 4.4. Strengths and Limitations

The main strength of this study is the large number of community-setting patients analyzed, which reflects the clinical reality of a clinical nutrition consultation. Another strength of this study is the use of a deep learning-based AI application to evaluate muscle ultrasonography, which reduced the interobserver variability in the assessment of muscle mass and, especially, muscle quality with an algorithm based on every single image. The main limitation of this study is the variability in the pathologies that can interfere with the extrapolation of data because they may have some bias related to the distinctive features of every pathology. Another limitation is the differences observed in inflammation related to age, but we have tried to control this condition with a multivariate analysis. The lack of cut-off values for any muscle quality variables made us use the median of the sample to establish the relation between muscle impairment and inflammation. On the other hand, the single-center nature of this study may affect the data’s reliability, while the involvement of professionals conducting the probes could impact the representativeness of the data. Finally, due to the cross-sectional design, this study cannot establish whether inflammation causes muscle degradation or vice versa.

## 5. Conclusions

This study underscores the significant impact of inflammation on malnutrition and muscle health, emphasizing the need for precise assessments in these conditions.

Patients with inflammation showed reduced muscle area and thickness on ultrasonography, along with poorer muscle quality, characterized by a higher fat content and a lower muscle percentage in the region of interest (ROI).

Elevated C-reactive protein (CRP) levels were inversely correlated with muscle area and muscle percentage, while showing a direct correlation with fat percentage. Patients with higher inflammation levels faced an increased risk of low muscle mass and poor muscle quality, even after adjusting for age and sex, highlighting the detrimental effects of inflammation on muscle health.

AI-assisted ultrasonography provided detailed insights into muscle composition and quality, demonstrating its value as a precise tool for assessing the impact of inflammation on malnutrition.

Future research directions related to this study focus on assessing the impact of inflammation on muscle, as evaluated through ultrasonography, across various patient groups and age ranges. Additional studies could explore the relationship between the identified parameters and patient prognosis, as well as how these parameters change in response to medical nutrition therapy. Furthermore, AI-assisted ultrasonography, as a radiomics application, holds potential for developing innovative biomarkers to aid in diagnosing, monitoring, and treating malnutrition and sarcopenia.

## Figures and Tables

**Table 1:** Differences in body composition variables between sex. BMI: body mass index; TBW: total body water; ASMI: appendicular skeletal muscle index; SFT: subcutaneous fat thickness; RFMT: rectus femoris muscle thickness; RFMA: rectus femoris muscle area; MiT: muscle index; FATiT: fat index; NMNFiT: no muscle no fat index; CRP: C-reactive protein.

	TOTAL (*n* = 502)	MEN (*n* = 213)	WOMEN (*n* = 289)	*p*-Value
ANTHROPOMETRY				
BMI (kg/m^2^)	22.26 (4.84)	23.29 (4.51)	21.49 (4.94)	<0.01
Arm circumference (cm)	24.39 (3.72)	25.40 (3.19)	23.59 (3.92)	<0.01
Calf circumference (cm)	31.62 (4.10)	32.26 (4.25)	31.14 (3.93)	<0.01
BIOIMPEDANCIOMETRY				
Resistance (ohm)	597.97 (111.08)	549.04 (94.91)	634.08 (108.42)	<0.01
Reactance (ohm)	51.57 (12.21)	49.72 (11.39)	52.93 (12.62)	<0.01
Phase Angle (°)	4.97 (0.98)	5.21 (0.99)	4.79 (0.94)	<0.01
TBW (l)	9.12 (3.05)	11.67 (2.39)	7.24 (1.92)	<0.01
ASMI (kg/m^2^)	5.99 (1.06))	6.68 (0.93)	5.49 (0.84)	<0.01
RECTUS FEMORIS MUSCULAR ULTRASONOGRAPHY				
SFT (cm)	0.78 (0.46)	0.54 (0.28)	0.97 (0.48)	<0.01
RFMT (cm)	0.98 (0.29)	1.06 (0.32)	0.91 (0.26)	<0.01
RFMA (cm^2^)	3.07 (1.16)	3.51 (1.25)	2.75 (0.96)	<0.01
MiT (%)	47.40 (11.39)	49.40 (12.57)	45.92 (10.22)	<0.01
FATiT (%)	38.68 (7.49)	37.78 (8.34)	39.34 (6.75)	0.02
NMNFiT (%)	13.92 (5.48)	12.82 (5.63)	14.74 (5.23)	<0.01
Pennation Angle (°)	5.19 (2.89)	5.61 (3.03)	4.85 (2.75)	<0.01
FUNCTIONAL MARKERS				
Handgrip Strength (kg)	21.49 (9.31)	26.44 (9.23)	17.86 (7.53)	<0.01
BIOCHEMICAL BIOMARKERS				
CRP (mg/L)	2.19 (1–7.8)	2.6 (1.10–7.14)	1.9 (1–7.93)	0.51
Albumin (g/dL	4.36 (2.37)	4.41 (2.44)	4.32 (2.31)	0.66
Prealbumin (g/dL)	22.43 (8.47)	22.78 (7.24)	22.17 (9.28)	0.47
CRP/prealbumin	0.09 (0.05–0.38)	0.11 (0.05–0.39)	0.08 (0.05–0.38)	0.24
CRP/albumin	0.51 (0.24–1.95)	0.6 (0.26–1.77)	0.42 (0.23–2.15)	0.21

**Table 2:** Differences in body composition depending on the degree of inflammation. BMI: body mass index; TBW: total body water; ASMI: appendicular skeletal muscle index; SFT: subcutaneous fat thickness; RFMT: rectus femoris muscle thickness; RFMA: rectus femoris muscle area; MiT: muscle index; FATiT: fat index; NMNFiT: no muscle no fat index.

	No Inflammation (*n* = 277)	Mild Inflammation (*n* = 115)	Moderate Inflammation (*n* = 92)	Severe Inflammation (*n* = 18)	*p*-Value
Sex (M/F) (%)	40.4/59.6	47.8/52.2	43.5/56.5	33.3/66.7	0.48
Age (years)	61.27 (16.89)	65.55 (13.27)	66.61 (15.69)	75 (7.81)	<0.01
ANTHROPOMETRY					
BMI (kg/m^2^)	21.73 (4.80)	23.31 (4.14)	22.28 (5.62)	23.68 (4.16)	0.02
Arm circumference (cm)	24.1 (3.61)	25.67 (3.95)	23.75 (3.67)	23.48 (2.24)	<0.01
Calf circumference (cm)	31.59 (4.19)	32.14 (3.95)	31.05 (3.89)	31.53 (4.59)	0.30
BIOIMPEDANCIOMETRY					
Resistance (ohm)	607.85 (115.34)	593.34 (103.84)	580.81 (106.82)	560.71 (96.61)	0.09
Reactance (ohm)	53.05 (11.79)	52.11 (12.32)	47.65 (12.29)	44.77 (11.73)	<0.01
Phase Angle (°)	5.04 (0.98)	5.07 (0.97)	4.71 (0.95)	4.56 (0.85)	<0.01
TBW (l)	9.04 (2.72)	9.42 (2.72)	9.18 (3.46)	8.19 (2.09)	0.39
ASMI (kg/m^2^)	5.91 (1.08)	6.15 (0.95)	6.03 (5.82)	6.14 (0.85)	0.21
RECTUS FEMORIS MUSCULAR ULTRASONOGRAPHY					
SFT (cm)	0.78 (0.44)	0.82 (0.52)	0.69 (0.38)	0.89 (0.57)	0.19
RFMT (cm)	1.01 (0.30)	0.96 (0.27)	0.91 (0.29)	0.89 (0.29)	0.02
RFMA (cm^2^)	3.19 (1.19)	3.01 (1.07)	2.85 (1.14)	2.68 (1.11)	0.03
MiT (%)	49.10 (12.19)	45.79 (9.94)	45.67 (9.55)	40.48 (11.57)	<0.01
FATiT (%)	37.58 (7.91)	39.77 (6.41)	39.76 (6.30)	40.47 (11.58)	<0.01
NMNFiT (%)	13.33 (5.63)	14.44 (5.32)	14.57 (4.97)	16.42 (5.74)	0.03
Pennation Angle (°)	5.17 (2.72)	5.09 (3.35)	4.68 (2.95)	6.38 (2.39)	0.51
FUNCTIONAL MARKERS					
Handgrip Strength (kg)	20.75 (9.54)	22.94 (9.21)	21.69 (8.54)	22.44 (9.49)	0.20

**Table 3:** Correlations between inflammation markers, phase angle, total body water, and ultrasonography variables. TBW: total body water; SFT: subcutaneous fat thickness; RFMT: rectus femoris muscle thickness; RFMA: rectus femoris muscle area; MiT: muscle index; FATiT: fat index; NMNFiT: no muscle no fat index.

	SFT (cm)	RFMA (cm^2^)	RFMT (cm)	Pennation Angle (°)	MiT (%)	FATiT (%)	NMNFiT (%)
CRP (mg/L)	r = 0.02; *p* = 0.68	r = −0.09; *p* = 0.04	r = −0.09; *p* = 0.05	r = 0.06; *p* = 0.19	r = −0.16; *p* < 0.01	r = 0.16; *p* < 0.01	r = 0.12; *p* < 0.01
CRP/prealbumin	r = 0.03; *p* = 0.58	r = −0.64; *p* = 0.16	r = −0.06; *p* = 0.18	r = 0.05; *p* = 323	r = −0.18; *p* < 0.01	r = 0.19; *p* < 0.01	r = 0.12; *p* < 0.01
CRP/albumin	r = 0.02; *p* = 0.75	r = −0.08; *p* = 0.09	r = −0.07; *p* = 0.09	r = 0.05; *p* = 0.29	r = −0.18; *p* < 0.01	r = 0.18; *p* < 0.01	r = 0.12; *p* < 0.01
Phase angle (°)	r = −0.05; *p* = 0.29	r = 0.46; *p* < 0.01	r = 0.44; *p* < 0.01	r = 0.19; *p* < 0.01	r = 0.29; *p* < 0.01	r = −0.22; *p* < 0.01	r = −0.31; *p* < 0.01
TBW (l)	r = −1.59; *p* < 0.01	r = 0.45; *p* < 0.01	r = 0.45; *p* < 0.01	r = 0.18; *p* < 0.01	r = 0.14; *p* < 0.01	r = −0.08; *p* = 0.08	r = −0.19; *p* < 0.01

**Table 4:** Risk factors of muscle mass parameters. LowMMUSArea (low muscle mass by area in ultrasonography); low MMUSthickness (low muscle mass by thickness in ultrasonography); CRP: C-reactive protein.

	OR	IC 95%	*p*-Value
LowMMUSArea (Male < 3.5 cm^2^; Female < 2.7 cm^2^)			
Sex	0.91	0.62–1.32	0.61
Age > 65 years	3.04	2.09–4.41	<0.01
CRP > 3	1.59	1.10–2.31	0.01
LowMMUSthickness (Male < 1.06 cm; Female < 0.9 cm)			
Sex	0.93	0.64–1.35	0.69
Age > 65 years	3.34	2.37–4.99	<0.01
CRP > 3	1.23	0.85–1.78	0.28

**Table 5:** Risk factors of the muscle quality parameters. LowMiT (low muscle index in ultrasonography); HighFATiT (high fat index in ultrasonography); CRP: C-reactive protein.

	OR	IC 95%	*p*-Value
LowMiT (Male < 47%; Female < 44%)			
Sex	0.89	0.62–1.31	0.57
Age > 65 years	2.18	1.49–3.18	<0.01
CRP > 3	1.25	1.01–1.54	0.04
Sarcopenia	1.72	1.13–2.62	0.01
HighFATiT (Male > 36%; Female > 36%)			
Sex	0.73	0.51–1.06	0.09
Age > 65 years	1.54	1.06–2.24	0.02
CRP > 3	1.19	0.97–1.46	0.09
Sarcopenia	1.26	0.83–1.89	0.28

## Data Availability

Data are contained within the article.
